# Advanced Therapies for Inflammatory Bowel Disease: Navigating Payor and Financial Challenges

**DOI:** 10.1007/s11894-024-00916-w

**Published:** 2024-01-20

**Authors:** Natalie Whitmire, Michelle Schlueter, Melissa Kirkpatrick

**Affiliations:** Division of Gastroenterology, UC San Diego Health, 9350 Campus Pt Dr Ste 2B, La Jolla, CA 92037 USA

**Keywords:** Medication prior authorization, Medicare, Biologics, Inflammatory bowel disease, Inflammatory bowel disease therapies, appeal process

## Abstract

**Purpose of Review:**

In the United Sates the cost of managing Crohn’s disease and ulcerative colitis, the two most common inflammatory bowel diseases, is a major factor that can alter the course of treatment. The increasing use of advanced therapies such as biologics and oral small molecules is a driver of these costs. Many IBD providers find navigating the payor and non-insurance cost assistance processes to be a significant challenge in care management. We aim to clarify these processes and provide an outline for success.

**Recent Findings:**

Insurance companies use various processes to manage medication costs and while they may not ultimately be cost-effective, the processes have continued and are increasingly complex. This complexity has led to measurable delays in care and negative outcomes.

**Summary:**

With a deeper understanding of payor and non-insurance cost-assistance processes we have developed a workflow for navigating the use of advanced therapies in the treatment of IBD.

**Supplementary Information:**

The online version contains supplementary material available at 10.1007/s11894-024-00916-w.

## Introduction

Crohn’s disease (CD) and ulcerative colitis (UC) are the two most common inflammatory bowel diseases (IBDs). These are chronic conditions marked by inflammation of the gastrointestinal (GI) tract that affect more than 1.5 million people in North America [1]. The causes are multifactorial but involve dysregulation of the immune system [2]. People with IBD will experience periods of exacerbation and remission with treatments focused on achieving and maintaining longer periods of remission. Advanced therapies for IBD, including infused and self-administered biologics and oral small molecules, target various immune system pathways. These therapies may help people achieve and maintain remission but come with financial considerations.

The cost of providing healthcare is substantially higher in the United States of America than in any other high-income country [3–5]. Although more recently skewed by pandemic-associated spending, in 2016 the US spent 17.7% of Gross Domestic Product (GDP) on healthcare while the next closest high-income country spent 12.4% [4]. Administrative costs, labor costs, and prices of services and goods are often cited as the drivers of this spending [4–6].

In 2016, IBDs were cited to have direct and indirect costs of 14.6 to 31.6 billion dollars annually [7]. Direct costs are led by inpatient hospitalization, emergency department visits, and pharmaceuticals though some estimates propose that pharmaceuticals will begin to top the list [8, 9]. This has added to the financial toxicity that patients with IBD face. A 2021 study found that in adult patients with IBD in the US, 23% reported financial hardships specifically due to medical bills and 16% reported cost-related medication non-adherence (skipping/delaying doses) [8].

The high cost of pharmaceuticals, in addition to several other healthcare forces, have created an environment where payors (insurance companies for purposes of this review) have implemented cost-saving measures. For providers taking care of those with IBD, these measures are often confusing and frustrating. A recent review found that when a PA is denied, 37% of those prescriptions are abandoned, a term that means the prescription was not picked up or shipped to the end user [10]. Providers need tools to help navigate these issues. While there is no simple answer to get every patient the preferred medication, there are strategies and processes that can improve the success rate of patients attaining necessary therapies. Three important considerations for medication access are the payor type, the insurance process,and non-insurance based cost assistance programs.

## Advanced Therapies

Advanced therapies is a term that applies to biologics and small molecule oral medications that are typically used in moderate to severe forms of CD and UC [Supp 1]. These therapies target several immune pathways that play a role in the immune dysregulation that occurs in these diseases. Small molecule oral medications are dosed once to twice daily. Biologics are large molecules administered via intravenous (IV) and/or subcutaneous routes with variable dosing frequencies. Some biologics are available as the reference product and as biosimilars; biologics that have no clinically meaningful difference from the reference product [11]. Many of these advanced therapies, dosage forms, or biosimilar availability did not exist five years ago. The widening landscape of therapy options can be beneficial for people with CD and UC while also introducing complexities to an already complex process. Recommending an advanced therapy for a patient is the first step in the process but a patient cannot be successfully treated unless they can consistently access the medication. One of the main barriers to access is cost.

### Payors

Due to the high cost of advanced therapies, most people with IBD will need to use medical and/or pharmacy insurance (payors) benefits to access these medications.

Most advanced therapies used in IBD that are self-administered, such as oral and injectable medications, will be reviewed and paid for by the pharmacy benefit manager (PBM). These are typically dispensed by a specialty pharmacy contracted with the payor. Most infusion therapies are reviewed and paid by medical insurance. Some are reviewed by the PBM but paid by the medical insurance. Others are reviewed and paid by the pharmacy benefits. Beyond this, there are still many ways that medications may be reviewed and paid for. The medical benefits and pharmacy benefits portions of insurance do not always communicate with each other, thus therapies that involve an infusion portion and self-administered portion will typically require a separate prior authorization for each.

Payors can be separated into non-government payor plans and government payors. Non-government includes any plan that does not receive government funding. Though a less inclusive term, these are often referred to as commercial plans. Government-funded plans include Medicare, Medicaid, Tricare, and any other plan that receives government funding. The distinction between funding is important as there are regulations prohibiting those with government-funded payors to receive copay savings cards and other services that may be offered by manufacturers or third parties. Once approved by the payor, advanced therapies under Medicaid and Tricare plans are often not cost-prohibitive. Medicare; however, warrants further discussion.

### Medicare

#### Basic Variations in Structure

Although Medicare has several distinct parts and many options for specific plans, the important focus for this article is Medicare Part B versus Medicare Part D. In addition to the majority of outpatient medical care, Part B covers injectable and infused drugs when these are administered by a licensed medical provider. Part D primarily covers medications administered by the patient, such as oral therapies and self-administered injectables (Table 1) [12].

**Table 1:** Summary of Medicare Part B and Medicare Part D for IBD therapies [13–16]

	Medicare Part B	Medicare Part D
Therapies	• Medications administered by a licensed medical provider • Drugs on Medicare’s SAD list excluded	• Self-injectables and oral therapies • Certain infusion providers will bill medications separately from infusion services
Coverage	All FDA-approved therapies • Off-label therapy, including dose-escalation, may be denied with option to appeal.	Subject to formulary requirements; may have preferred agents or impose step therapy restrictions
Cost	• Deductible • After deductible, 20% co-insurance applies • Patients with a supplement, which covers the 20%, will generally have no copay • Cost for any off-label therapy that is not approved is significant	• Deductible • 25–33% copayment per fill after deductible met • Once trOOP is reached for covered drugs, patient enters catastrophic coverage phase and no longer has copays

### Coverage and Cost: Infusion Medications

Infusion therapies are covered by Medicare for all FDA-approved indications. Standard Medicare Part B does not require prior authorization for these indications. Off-label usage (including dose escalation) will be subject to infusion center policies and procedures and risk tolerance in the case that a claim is later denied. Under Medicare Part B, patients are responsible for meeting a deductible, after which they will pay up to 20% for each Medicare-covered service or drug. Most patients opt for a Medicare supplement, which will cover the share of cost that Part B does not cover, minus any deductibles or out-of-pocket (OOP) maximums [12]. Using the additional Medicare supplement, patients typically will not have a copay for infusions. Given the relative lack of limitations on coverage, as well as the minimal OOP costs for most infusions, infused therapies are generally financially preferable for patients with Medicare.

### Coverage and Cost: Self-Administered Medications

When infusion therapies are not appropriate, Medicare Part D will be the responsible payor for prescriptions. In 2023, there were 801 Medicare Part D plans, with 20–30 different plans available in each state. Each plan has its own formulary and step therapy requirements. In addition to having more restrictions on therapy options, patients often have greater share of cost with Medicare Part D. When using the prescription drug benefit, patients must first meet a deductible; the amount of this deductible is variable, but the standard deductible is higher than the Part B/medical supplement deductible. Once the deductible has been met, patients will pay a 25–33% copayment for each fill of their prescription medication until they reach a certain amount of true OOP drug spending. True OOP spending (often referred to as trOOP) encompasses patient’s out-of-pocket cost as well as payments made by certain assistance programs and the value of manufacturer discounts provided under the Medicare coverage gap discount program [13, 14]. Once trOOP reaches $8000, the patient enters catastrophic coverage where they will no longer have a copay. Annual costs under this structure can be significant, and many patients may have difficulty paying hundreds to thousands of dollars at a time for a copayment. However, the Inflation Reduction Act passed in 2022 has several Medicare reforms that will reduce patient cost in the years to come, including implementing a cap of $2000 in patient OOP costs in 2025 [15, 16].

### Additional Medicare Considerations

Not all patients over 65 will be covered by Medicare; additionally, it is possible for patients to have Medicare A/B for medical coverage but have a non-government plan for prescription drug coverage. Copay savings cards are not available to patients with government-funded insurance, such as Medicare. If, however, patients have a Medicare medical coverage but non-government prescription coverage, copay savings cards can usually be used for any self-administered medication.

Certain plans, known as Medicare Advantage Plans, are available through private companies who are paid by Medicare for the coverage they provide. Medicare Advantage Plans may have different premiums, cost sharing, and access restrictions than original Medicare plans, but these should not be confused with private, non-Medicare insurance [17].

Although infusion therapies are generally financially preferable for patients with Medicare, cost structures at infusion centers are not always straightforward. Some infusion centers will separate administration and drug costs for infusions, billing the former to Part B and the latter to Part D. When this occurs, patients can have high copays for infused medications similar to what they would expect with self-administered drugs. Similarly, though you may consider administering injectables at an infusion center with the intention of billing to the Part B medical benefit, this practice is prohibited. Medicare maintains a‘Self-Administered Drugs’ (SAD) Exclusion List, which delineates which drugs are not covered by Medicare Part B, even if administered by a health care professional [18]. This effectively prohibits the administration of selected drugs in a medical setting in an effort to avoid high copays experienced with Medicare Part D. For example, many patients on Medicare received ustekinumab subcutaneous doses at infusion centers, which carried little to no cost when billed under the Part B medical benefit. However, when the SAD list became effective for this medication, patients had to transition to filling ustekinumab through their Part D plan, leading to thousands of dollars in new OOP costs that necessitated therapy changes for some patients.

## Insurance Process

As we have stated, the high cost of advanced therapies means that people with IBD will rely on payors to afford medications. It also means these medications are subject to a review process by payors. This insurance-related review process is called prior authorization (PA).

### Prior Authorizations

A review article from 2019 outlines the basic process: provider answers clinical questions and payor responds based on criteria that should correspond to published data and guidelines [19]. In practice, the process is more complicated [20]. Payors use variable forms, variable criteria, and variable third parties to complete the review process. A review of 50 of the top 125 insurance plans by market share in 2017 found that 98% were inconsistent with the American Gastroenterology Association (AGA) guidelines for ulcerative colitis and 90% were inconsistent with the AGA Crohn’s disease treatment pathway [21]. Despite this, one study found that 93.4% of specialty medications are eventually covered and filled. It is important to note that the medications were primarily for on-label indications and doses [22].

Despite inefficiencies, the process is not going away soon. A recent review noted “PA requirements [from 2007 to 2019]…for immune disease therapies…have increased from 35–67%. There is a lack of evidence indicating cost-savings associated with the current PA system” [23]. One study found that the average time from agreeing to start a biologic to payor approval was 30.5 days [24]. Another study found similar results but furthermore demonstrated that PA requirement negatively impacts patient outcomes. They showed that from the time an advanced therapy was recommended, the need for PA increased the likelihood of IBD-related healthcare utilization within 180 days by 12.9% and corticosteroid dependence at 90 days by 14.1% [25].

Obtaining PA involves initial PA submission and may also involve several rounds of appeals. It is helpful to start with understanding the terminology (Table 2).

**Table 2:** Glossary of Terms

Term	Definition
Formulary	List of covered medications
Pharmacy Benefit Manager (PBM)	A third party used by an insurance company to negotiate with manufacturers and often review prior authorizations for medications and other medication use activities
Deductible	Amount of money a person must spend before certain healthcare benefits start
Co-payment	The amount of money a person pays after their insurance benefit has been applied
Co-insurance	Typically a % owed by a patient for a particular good or service (as opposed to a set fee)
Prior authorization (PA)	Process that requires providers to submit information before payors will provide coverage of a product or service
Step edit/step therapy	Treatment(s) tried and failed prior to another treatment being approved
Quantity limit	Amount of drug/ time permitted Example: 1 syringe per 56 days
Site of Care optimization	Re-direction of services to a (typically) lower-cost setting (to payor, not necessarily the patient)
Appeal	Formal process of requesting reconsideration of a decision made by the payor
Peer to Peer	Provider to provider discussion (typically a phone call) that may or may not be part of the appeals process whereby the prescribing provider discusses rationale for use of a medication to a provider employed by the insurance company
Independent Medical Review or External Review	Process required by law that allows for review of a request outside of the payor. Typically occurs after internal appeals are exhausted. Completed by the state or an independent contracted company
Bridge Program	Manufacturer related term. This is a temporary, free supply of medication provided to a patient with commercial insurance when they are awaiting payor approval or have a gap in medication access. Programs may go by various names.
Co-pay savings programs	Offered by manufacturers and independent groups (e.g. Patient Assistance Fund, Patient Access Network (PAN) Foundation, etc.). Free programs for those with non-government insurance to assist with copay costs. Medication must be covered by insurance and then copay card can apply. Typically has an annual limit.
Patient assistance programs	Offered by manufacturers and independent groups (e.g. Patient Assistance Fund, Patient Access Network (PAN) Foundation). Includes government and non-government insurance and uninsured. Medication is provided free to the person. Typically has financial qualifications required
Copay accumulator	Program that payors use to limit how manufacturer contributions count towards deductibles and out of pocket spending. Manufacturer coupon is used until funds run out and then patient begins to spend toward deductible and out of pocket
Copay maximizer	Program that payors use to limit how manufacturer contributions count towards deductibles and out of pocket spending. The value of the manufacturer coupon is spread evenly across the year
Buy and bill	Process where medication is purchased by the same provider that administers the medication and then bills insurance for the medication and administration costs
Brown bagging	Process by which a medication is shipped from a pharmacy to a patient directly and the patient then brings the medication to a provider office or infusion center for administration or the medication is administered in their home
White bagging	Process by which a medication is shipped from a pharmacy directly to a provider office or infusion center for patient administration

The majority of initial PAs will ask the following (often in checklist yes/no format):

- Diagnosis: this should be clear (where possible) and written in terms that match guideline terminology.
- Labs: for most advanced therapies, this includes up to date tuberculosis and hepatitis B serologies.
- Dose and quantity per number of days being requested.
- Current symptoms.
- Relevant scoring tools.
- Past tried and failed medications.

### Appeals

Once an initial PA is denied, there are typically several next steps. All payors will have an appeals process. These processes can include peer to peer discussion, submission of appeal letters, second level appeals after initial appeal denial, and an external review process (either done by the state or a third party but required for all plans). Peer to peer discussion may offer a quicker resolution as they can often be scheduled and completed within seven days. Some payors do not offer peer to peer or only offer it at specific times such as before or after an appeal has been filed. Appeal letters can be marked urgent which are typically reviewed within 72 h but the payor will decide if they meet criteria for urgent review. The standard review time for most payors for appeal letters is 30–45 days. The external review process is also typically 30–45 days.

Reasons for denial are widespread. Sometimes simple information was missed, while other times a complicated case needs to be laid out. Payors use many automated criteria for initial prior authorizations that do not often allow for explanations. For infusion medications, it is common to have “site of care optimization” where the payor requires a specific infusion administration site based on cost/contracting. These often seem to be denials when they deny the place of service, not the medication or service itself. Depending on your payor mix you may see this very often.

### Approvals

Once a medication is approved, at any step in the process, the prescription should be sent to the in-network, payor-preferred specialty pharmacy where applicable. Many payors will list this on the authorization. For those who are commercially insured, the patient should provide the copay savings card information to the specialty pharmacy. For those with government-sponsored prescription insurance, the patient should reach out to the specialty pharmacy to determine their copayment. If the copayment is not affordable, the patient can pursue a patient assistance program from the manufacturer or a third-party organization.

Infusion therapies provided via a specialty pharmacy will follow the same approval process. For infusion therapies provided via buy and bill, once approved, the infusion center will proceed with the infusion appointment. Billing for the medication and administration, including copay savings programs, will take place after the service is provided.

As a patient case becomes more complicated, has fewer medication therapy options, and necessitates unique doses and combinations of therapies, we often follow the same process as outlined above knowing that we will be in an appeal process or relying more on support outside of insurance. Fortunately for people with IBD, insurance is not the only process involved in acquiring necessary medications. There are several support programs available that work with or independently from insurance.

## Non-Insurance Programs

The majority of non-insurance programs come in the form of manufacturer-related copay savings programs and patient assistance programs. These serve as a safety net to improve patient access to medications. According to IQVIA, a healthcare analytics company, copay savings programs decreased patients’ out-of-pocket costs by $19 billion in 2022 and $80 billion over the previous 5 years [26]. Copay card utilization rates are approximately 14% for all brand name medications. However, when looking at specialty drugs, utilization rates increase to 50% for non-government insured patients [27]. Patient assistance programs are more difficult to track as they are not usually included in pharmacy claims data. Including all payor types, it is estimated that up to 42% of all branded specialty prescriptions utilize copay or patient assistance programs [28].

### Manufacturer-Related Copay Savings Programs

Copay savings programs are offered by manufacturers for brand name medications and are designed to help offset out-of-pocket costs for non-government insured patients, often reducing a copay to $5. Patients with government-sponsored health plans such as Medicare, Medicaid, or Tricare are ineligible due to federal anti-kickback statutes. Of note, copay savings programs are prohibited in some states if a generic version of the branded medication is available. This does not apply to biosimilars because they are not considered generics, which means copay cards can be used in this instance if available for either the reference product or their corresponding biosimilar(s). Copay programs can be accessed through the medication or manufacturer websites and obtained by the patient or health care provider. Assistance is provided through issuance of a copay card or coupon and are typically processed similar to insurance at the point of sale at a specialty pharmacy or infusion center. Lastly, it is important to note that there may be monthly or yearly spending limits associated with these programs.

While these copay savings programs are meant to help offset out-of-pocket costs for patients, payors are making this more difficult through copay accumulator and maximizer programs associated with some plans. Both copay accumulator and maximizer programs shift drug costs to manufacturers and patients by maximizing spending limits on copay cards/coupons and excluding copay assistance dollars towards a patient’s deductible or out-of-pocket maximum. Some state’s laws prohibit these types of programs.

### Patient Assistance Programs

Most manufacturers have patient assistance programs (PAP) which are based on income eligibility criteria that can provide medication at little to no cost to patients whose insurance denies a medication or for those who are uninsured or underinsured. Each manufacturer program has different eligibility requirements and guidelines for assistance. Both provider and patient must complete an application, which is usually found on manufacturer websites and must be renewed annually. Some manufacturers do allow for e-submission and others require faxing of application. A patient may be asked to submit additional documentation that includes (but is not limited to): insurance card copies, proof of income, Explanation of Benefits (EOB) statement that shows out-of-pocket-costs for the current year (Medicare Part D patients). In the case of Medicare Part D, patients may need to attest that a certain percentage of their income goes towards medications and/or medical costs.

In addition to manufacturer-sponsored patient assistance programs, there are also state and non-profit programs to assist patients financially. Non-profit organizations such as: The Patient Access Network (PAN) Foundation, National Organization for Rare Disorders (NORD), HealthWell Foundation, and others all offer patient assistance programs to eligible individuals. These programs act similarly to manufacturer-based programs and will have income eligibility requirements and specific guidelines for assistance. Funds can open and close throughout the year based on money received by the foundation but are often set for the year and exhausted before year’s end, so applying early can be helpful.

### Other Funding

One last option that is available to patients outside of copay and patient assistance programs are third-party discount websites and discount pharmacies. The pricing of medications can vary at different pharmacies and these options may be helpful, particularly for medications typically used for mild forms of disease. Unfortunately, out-of-pocket costs are still high for advanced therapies in the treatment of IBD, even when discounts are provided. The influx of biosimilars coming to market may offer significant savings and these cost programs may be a more viable option for patients in the future.

## Process for Success

Obtaining PA and the subsequent steps reviewed in this article is time-consuming and a provider cannot bill for this time; as such, it is critical to take advantage of efficient, reproducible processes and systems to minimize wasted time [9].

Ideally, a practice should have one organized, dedicated person(s) responsible for completing PAs. This does not need to be a licensed person so long as they are trained in the terminology and process and willing to follow through. To support this person (or people), it is important to put procedures in place to collect pharmacy benefits information, not just medical benefits, and to set aside dedicated time. Follow-up phone calls often have long hold times. There needs to be an opportunity for multi-tasking that allows the person to remain on hold. This person should be given tools to document and track where they are in the process and to keep records of the denials, approvals, and appeal letters. Programs are available that can help streamline the PA process. Some of these programs exist as their own stand-alone website/portal or can be integrated within an electronic medical record (EMR). This can reduce administrative time by automatically entering patient, provider, and payor information. Some of these programs do not have associated fees for providers.

**Fig. 1:**
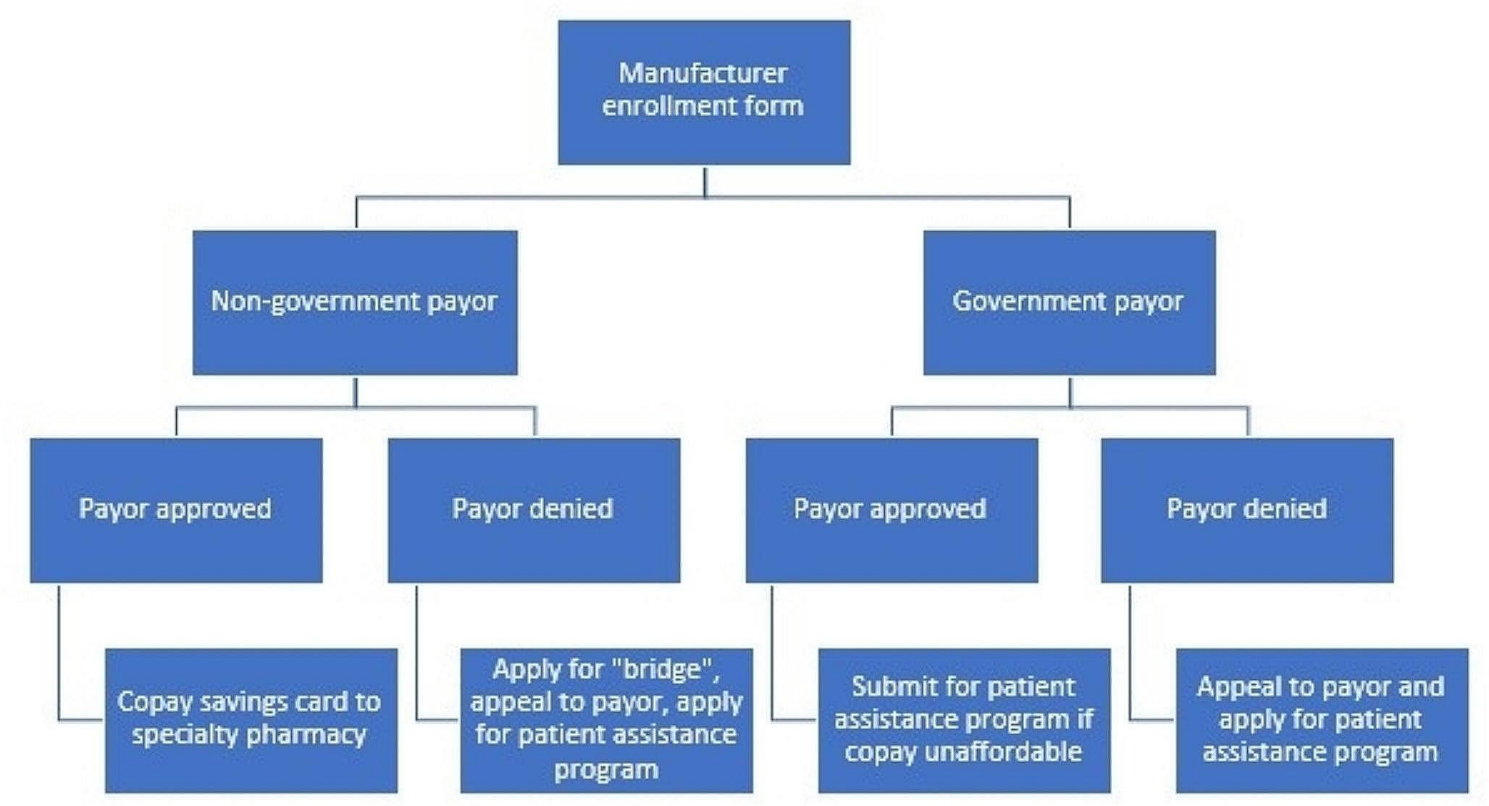
The interaction between hub services and the prior authorization process

Most manufacturers offer “hub” services which serve to act as an intermediary between all stakeholders: the payor, patient, provider, specialty pharmacy, and the manufacturer. Hub services can offer benefits investigations, PA processing information, copay savings programs, financial assistance, patient education, adherence programs, administration support, and, in some cases, medication. These hubs help in the complicated process of getting a specialty drug to the patient. We recommend enrolling a patient in hub services, if the patient consents, at the same time as initiating the PA (Fig. 1). This enrollment can be mostly completed by non-clinical staff and patient consent may be done electronically in many instances.

A different team may process PAs for infused medications compared to injectable and oral medications, such as an infusion center intake team. Infusion centers will typically require the infusion orders; recent chart notes; patient demographics including height, weight, and allergies; patient contacts including phone and address; insurance information; and annual tuberculosis and hepatitis B serologies, regardless of therapy. In our practice we have created templates for documentation that automatically inserts this data into a single note to cut down on the number of faxes and to ensure information is not missed that would cause delays [Supp 2]. We have found it useful to have internal contacts at various infusion companies outside of our health system to help minimize treatment delays. This is particularly useful when we receive a denial for “site of care” and need to transition to an alternate infusion setting when a person is already established on a therapy.

As the clinician, there are things you can and must do to make this process successful. Setting patient expectations for this process, the time involved, and the potential for changing to alternative therapy options when appropriate choices exist will alleviate patient stress. Aside from this reassurance and education, the most important thing you can provide is detailed documentation. Having the needed initial PA information clearly documented in your notes will increase efficiency when submitting for PA. Your notes will optimally contain the timeline of past medication failures (including any dose escalation) and reasons for failure of the therapy (primary nonresponse, secondary loss of response, adverse drug events). You may want to list treatments that have not been tried but are inappropriate for any standout reasons. Imaging, procedures, and biochemical markers like fecal calprotectin and C-reactive protein will further support requests though are often not part of the initial PA.

When an initial PA is denied there are several options for appeal and/or peer-to-peer as we have detailed. The task for the clinic point person is finding out what options are available and how to complete or submit them. The task for the clinical staff is creating easy-to-use appeal letter templates. The Crohn’s and Colitis foundation has several of these available [15]. In our center we create appeal letters within a patient EMR record so that they become part of the medical records and can be found and used again when the medication needs reauthorization and appealed again in the future.

As there are many steps to this process, our center has found it useful to document the steps and updates within one encounter, adding new information as it becomes available.

Lastly, this process can be stressful for people with IBD. Keeping a patient updated and informed at each step, including expected time for each step to resolve, will help. This can be mostly done with automated messaging or pre-made phrases.

## Conclusion

Patients with IBD face substantial financial toxicity which increases as they require advanced therapies. Navigating the payor and financial challenges surrounding these therapies requires understanding the payor types, insurance process, and the non-insurance based programs available in addition to developing workflows to manage this within your practice. Here we have provided a review of the current insurance process and best practices that help our center obtain necessary treatments for our patients with IBD.

### Electronic Supplementary Material

Below is the link to the electronic supplementary material.

Supplementary Material 1

Supplementary Material 2
