# Supplementary material for: Advanced Therapies for Inflammatory Bowel Disease: Navigating Payor and Financial Challenges
Source: Curr Gastroenterol Rep. 2024 Jan 20;26(3):68–76. doi: 10.1007/s11894-024-00916-w (PMC10937800; doi:10.1007/s11894-024-00916-w)
Supplement: Supplementary file 1 — Supplementary Material 1 [file 11894_2024_916_MOESM1_ESM.docx]

| **Medication** | **Mechanism** | **Route: Induction** | **Route: Maintenance** | **Biosimilar(s) available in US** |
| --- | --- | --- | --- | --- |
| infliximab | Anti- tumor necrosis factor (TNF) | IV | IV, SubQ | yes |
| adalimumab |  | SubQ | SubQ | yes |
| certolizumab |  | SubQ | SubQ | no |
| golimumab |  | SubQ | SubQ | no |
| vedolizumab | Anti-integrin | IV | IV, SubQ | no |
| natalizumab |  | IV | IV | no |
| ustekinumab | Anti- interleukin 12/23 | IV | SubQ | no (pending with FDA) |
| risankizumab-rzaa | Anti- interleukin 23 | IV | SubQ | no |
| mirikizumab-mrkz |  | IV | SubQ | no |
| tofacitinib | Janus kinase (JAK) inhibitor | PO | PO | n/a |
| upadacitinib |  | PO | PO | n/a |
| ozanimod | Sphingosine 1-phosphate (S1P) receptor modulator | PO | PO | n/a |
| etrasimod |  | PO | PO | n/a |

IV: intravenous; SubQ: subcutaneous; PO: oral
