# Supplementary material for: Advanced Therapies for Inflammatory Bowel Disease: Navigating Payor and Financial Challenges
Source: Curr Gastroenterol Rep. 2024 Jan 20;26(3):68–76. doi: 10.1007/s11894-024-00916-w (PMC10937800; doi:10.1007/s11894-024-00916-w)
Supplement: Supplementary file 2 — Supplementary Material 2 [file 11894_2024_916_MOESM2_ESM.docx]

**Orders sent for initiation of new therapy to outside infusion center:**

**Name:**

**DOB:**

**Sex:**

**Allergies:**

**Phone:**

**Address:**

**Infusion Company:**

- **Medication:**
- **Diagnosis**:
- **Notes**:

**Insurance information:**

**Recent weights:**

**Quant TB**:

**Hepatitis B serologies**

Required intake information: (pick list)

Orders sent to provider for signature, will fax when completed

Signed orders

Most recent clinical notes

Relevant lab values

Most recent endoscopy report

other

This note and above items faxed to: (pick list)

(list of frequently used infusion groups and associated fax numbers)
